# Lanreotide autogel/depot in advanced enteropancreatic neuroendocrine tumours: final results of the CLARINET open-label extension study

**DOI:** 10.1007/s12020-020-02475-2

**Published:** 2020-10-14

**Authors:** Martyn E. Caplin, Marianne Pavel, Alexandria T. Phan, Jarosław B. Ćwikła, Eva Sedláčková, Xuan-Mai Truong Thanh, Edward M. Wolin, Philippe Ruszniewski, on behalf of the CLARINET Investigators

**Affiliations:** 1 Department of Gastroenterology and Tumour Neuroendocrinology, Royal Free Hospital, London, UK; 2 Department of Medicine, Division of Endocrinology and Diabetology, Universitätsklinikum Erlangen, Friedrich Alexander University Erlangen-Nürnberg, Erlangen, Germany; 3 Department of Hematology‐Oncology, University of Texas Health Science Center at Tyler, Tyler, TX USA; 4 Department of Cardiology and Cardiac Surgery, School of Medicine, University of Warmia and Mazury, Olsztyn, Poland; 5 Department of Oncology, First Faculty of Medicine and General Teaching Hospital, Prague, Czech Republic; 6 Medical Affairs, Ipsen Pharma, Boulogne-Billancourt, France; 7 Tisch Cancer Institute at Mount Sinai and Icahn School of Medicine at Mount Sinai, New York, NY USA; 8 Division of Gastroenterology and Pancreatology, Beaujon Hospital, Clichy, France; 9 Université de Paris, Paris, France; 10 Present Address: Cancer Treatment Centers of America at South Eastern Regional Center, Atlanta, GA USA; 11 Present Address: Diagnostic and Therapeutic Center – Gammed, Warsaw, Poland; 12 Present Address: Center for Carcinoid and Neuroendocrine Tumors, New York, NY USA

**Keywords:** Neuroendocrine tumours, Lanreotide autogel, Lanreotide depot, Safety, Progression-free survival

## Abstract

**Purpose:**

In the phase III CLARINET study (NCT00353496), lanreotide autogel/depot (lanreotide) significantly improved progression-free survival (PFS) vs placebo in patients with non-functioning intestinal or pancreatic neuroendocrine tumours (NETs). The aim of CLARINET open-label extension (OLE) (NCT00842348) was to evaluate long-term safety and efficacy of lanreotide in these patients.

**Methods:**

Patients from the CLARINET study were eligible for the OLE if they had stable disease (irrespective of treatment group) or progressive disease (PD) (placebo-treated patients only). All patients in the OLE received lanreotide 120 mg every 28 days. Computed tomography or magnetic resonance imaging scans were conducted every 6 months and assessed locally for PD (the final scan was also assessed centrally).

**Results:**

Overall, 89 patients took part in the OLE (lanreotide, *n* = 42; placebo, *n* = 47). Median (range) exposure to lanreotide in patients who received lanreotide in the core study and OLE (LAN–LAN group) was 59.0 (26.0–102.3) months. In this group, the overall incidences of adverse events (AEs) and treatment-related AEs were lower in the OLE than in the core study. Median [95% CI] PFS in the LAN–LAN group was 38.5 [30.9; 59.4] months. In placebo-treated patients with PD at the end of the core study, time to death or subsequent PD during the OLE was 19 [10.1; 26.7] months.

**Conclusions:**

This study provides new evidence on the long-term safety profile and sustained anti-tumour effects of lanreotide autogel/depot in indolent and progressive metastatic intestinal or pancreatic NETs.

## Introduction

CLARINET was a phase III, 96-week, placebo-controlled study evaluating the effect of lanreotide autogel (depot in the USA) 120 mg every 4 weeks in patients with metastatic grade 1 or 2 (Ki-67 < 10%), non-functioning intestinal or neuroendocrine tumours (NETs). Lanreotide autogel/depot treatment was associated with a significant improvement in progression-free survival (PFS) [1]. To date, CLARINET is the most comprehensive and robust study of the anti-tumour effects of a somatostatin analogue (SSA) in patients with metastatic enteropancreatic NETs, and based on the study, lanreotide autogel/depot was approved for this indication in the USA and Europe [2, 3].

Designed prior to these approvals, the open-label extension (OLE) of the CLARINET study offered lanreotide autogel/depot 120 mg (hereafter lanreotide) as an ongoing treatment option for patients with stable disease (SD) at the end of the 96-week core study treatment period (whether they received lanreotide or placebo during this period), as well as for patients who had progressive disease (PD) at any time, while receiving placebo in the core study. The primary objective of the CLARINET OLE was to evaluate the long-term safety of lanreotide administered every 4 weeks in patients who continued lanreotide from the core phase to the OLE (LAN–LAN group), as well as those who entered the OLE after receiving placebo (PBO–LAN group). Another objective was to further investigate the long-term efficacy of lanreotide in patients with enteropancreatic NETs—notably, the median PFS was not reached in lanreotide-treated patients in the core study [1]. Also, the OLE was designed to allow evaluation of anti-tumour effects in patients who switched from placebo to lanreotide. A pre-planned interim analysis of the OLE data (conducted on completion of the core study) demonstrated the acceptable long-term safety/tolerability of lanreotide, and showed continued anti-tumour effects in patients with SD, with a median PFS [95% CI] of 32.8 [30.9; 68.0] months [1]. This contrasts with a median of 18 months with placebo in the core study [1].

The CLARINET OLE study has now been completed and here we report the final safety and efficacy results, including an estimation of the time to subsequent PD in the PBO–LAN group who experienced PD, while receiving placebo in the core study and analyses of PFS in clinically relevant subgroups.

## Materials and methods

### Patients

Inclusion and exclusion criteria for the CLARINET core study and OLE have been published previously [1, 4]. In brief, patients in the core study were adults (≥18 years) with: well- or moderately differentiated, non-functioning NETs; tumours that were measurable according to Response Evaluation Criteria In Solid Tumours (RECIST, version 1.0) and with a Ki-67 < 10%; a primary tumour in the pancreas, small intestine, appendix, hindgut or unknown location; target lesion(s) classified on somatostatin-receptor scintigraphy as grade ≥2 (Krenning scale); metastatic disease or a locally advanced tumour that was inoperable or for which surgery had been refused; and a World Health Organization (WHO) performance status score ≤2. Patients at participating centres were eligible to take part in the CLARINET OLE if they had been treated in the core study, had centrally assessed SD (RECIST v1.0) at the end of the core study (regardless of the treatment to which they were initially randomised) or centrally assessed PD (RECIST v1.0), while receiving placebo in the core study. Patients’ WHO performance score also had to be ≤2. Patients could be withdrawn from the OLE if local assessments indicated tumour progression, for safety reasons, or at their own request.

Informed consent was obtained from all patients before enrolment into the OLE, prior to any study-specific procedures.

### Trial design and interventions

The phase III, multicentre CLARINET core study (NCT00353496) was conducted in the USA, India and 12 European countries between June 2006 and April 2013. The OLE was a single-arm (lanreotide), open-label study conducted in the USA, India and eight European countries between February 2009 and December 2015 (ClinicalTrials.gov: NCT00842348; EudraCT: 2008-004019-36) [4]. Patients were enroled within 4 weeks of their last study visit during the core study; all received lanreotide 120 mg by deep subcutaneous injection every 28 days until disease progression, death, early withdrawal or until lanreotide was approved for tumour control in their respective country. Patients could be withdrawn from the study for any reason, including adverse events (AEs), protocol violations, withdrawal of consent, loss to follow-up and disease progression or death.

Study documents were reviewed and approved by an Independent Ethics Committee/Institutional Review Board in each country before the start of the OLE. The study was conducted under the provisions of the Declaration of Helsinki [5], and in accordance with the International Conference on Harmonisation Consolidated Guideline on Good Clinical Practice [6]. The study also adhered to all local regulatory requirements. Protocol amendments after the start of the study are provided (see Supplementary Appendix).

### Safety assessments

Safety assessments conducted during the OLE included: AEs and treatment-related AEs; physical examination, vital signs (including electrocardiogram [ECG]) and clinical laboratory tests (every 24 weeks); and gallbladder ultrasonography (every 48 weeks). AEs were defined as undesirable medical conditions or the deterioration of a pre-existing medical condition following or during exposure to the pharmaceutical product administered in the study, whether or not considered causally related to the product. All AEs were coded according to the Medical Dictionary for Regulatory Activities (MedDRA®, version 18.1) preferred term and system organ class. The severity of AEs was defined as follows: mild, symptoms did not alter the patient’s normal function; moderate, symptoms produced some degree of functional impairment, but were not hazardous, uncomfortable or embarrassing to the patient; and severe, symptoms were definitely hazardous to wellbeing, significantly impaired function or incapacitated the patient. AEs were monitored from the time that the patient withdrew or completed CLARINET core study until withdrawal in CLARINET OLE, and were elicited by direct, non-leading questioning or by spontaneous reports. AEs that were ongoing at the end of the core study were recorded and followed up during the OLE.

### Efficacy assessments

Computed tomography or magnetic resonance imaging scans were performed every 24 weeks during the OLE, at the completion or withdrawal visit, and at any time in case of biological or clinical signs of PD; scans were assessed for signs of PD according to RECIST v1.0. All scans were assessed locally, but the scans from Visit 1 and the last visit, as well as any showing PD, were also reviewed centrally. The main efficacy endpoint was PFS in lanreotide-treated patients, defined as the time from randomisation in the core study to the first occurrence of PD or death in the core or OLE study, i.e., PFS data from OLE were appended to PFS data obtained in the core study, thereby extending the follow-up time for PFS. Additional efficacy endpoints were as follows: PFS in clinically relevant subgroups of lanreotide-treated patients (see *Populations and subgroups* below); time to death or subsequent PD in patients switching to open-label lanreotide after experiencing PD during placebo treatment in the core study [PBO (PD)–LAN group]; PFS in the PBO–LAN group who entered the OLE with SD [PBO (SD)–LAN group].

### Populations and statistical analyses

#### Populations and subgroups

The Safety population comprised all patients who received at least one dose of lanreotide in the OLE. The intent-to-treat (ITT) population for analysis of efficacy endpoints comprised all patients randomised in the core study (regardless of whether they continued into the OLE). The per-protocol (PP) population comprised all patients in the ITT population for whom no major protocol violations/deviations occurred during the core study (protocol violations/deviations in the OLE were not taken into account).

Several clinically relevant subgroups were defined before the start of core study according to: presence/absence of PD; prior/no prior therapy for NET; location of primary tumour (midgut/pancreas/hindgut/other); enrolment at centres within or outside the USA. Additional clinically relevant subgroups were defined *post-hoc* according to tumour grade (1/2) and hepatic tumour load (≤25%/>25%) at entry into the core study.

#### Statistical analyses

Summaries of demographic and disease characteristics at baseline and safety data were based on the Safety population according to the sequence of treatment received: lanreotide in both core and OLE studies (LAN–LAN) and placebo in core and lanreotide in OLE (PBO–LAN). AEs were evaluated by: combining OLE and core AE data within the LAN–LAN group (pooled); comparing OLE and core AE data within the LAN–LAN group (OLE vs core); and comparing OLE AE data according to treatment received in the core study (LAN–LAN vs PBO–LAN).

The main analysis of PFS was based on the ITT population, but was also repeated for the PP population. Time to event was described using Kaplan–Meier plots, presented in months (1 month approximated to 4 weeks).

It was anticipated that patients withdrawn from the OLE for locally assessed PD may subsequently be shown to have SD on central review. A pre-planned sensitivity analysis was therefore conducted to evaluate the potential impact of withdrawal of these patients on PFS estimates. A *post-hoc* sensitivity analysis of PFS was also performed to address potential selection bias due to patients with SD who completed the core study, but did not enter into the OLE. This analysis assumes that these patients had an event at the first scheduled radiological assessment in the OLE (i.e., at 24 weeks after the last assessment in the core study).

Statistical evaluation was performed using Statistical Analysis System® (SAS, version 9.3).

## Results

### Patients and treatment exposure

**Fig. 1:**
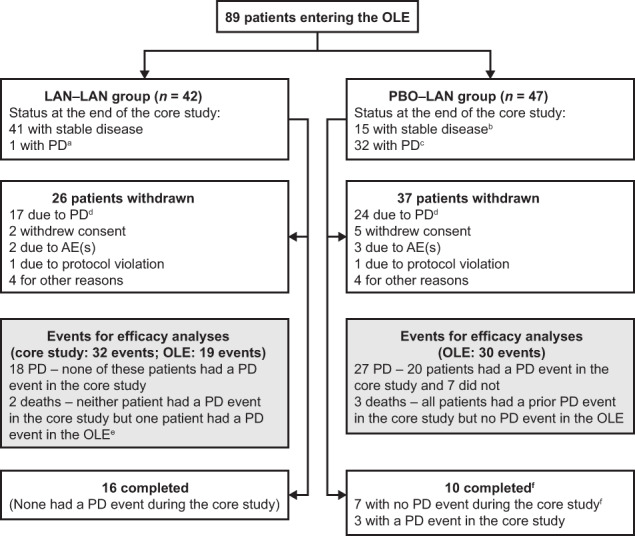
Flow of patients through the OLE. ‘Completed’ means the patient had had no PD events during the OLE at the time that the OLE was terminated. ^a^One patient was enroled by the investigator before centrally assessed PD was confirmed (the patient was withdrawn when the confirmation was received). ^b^Includes 1 patient who was withdrawn from the core study due to investigator judgement of PD but had an a posteriori central assessment of SD—this patient was then enroled in the OLE. ^c^Including 1 patient who was withdrawn from the core study due to a centrally assessed PD, but was erroneously classified as having SD at the time of the core study database lock—this subject was censored in the primary analysis of PFS in the core study, but was included as an event in the analysis of PFS in the OLE. ^d^Withdrawal from the OLE due to ‘PD’ did not always represent an event in the analysis of PFS: part-way through the study, the sponsor sent additional clarification to all sites on how to complete local tumour evaluations in order to have a standardised approach for the assessment of progression status across the study sites; all radiological scans that had already been evaluated were re-evaluated at this time. ^e^Only the PD event was included in the analysis of PFS. ^f^Includes 1 patient with PD at the final study visit. OLE open-label extension, LAN–LAN *group* patients receiving lanreotide autogel/depot in core study as well as the OLE study, PBO–LAN *group* patients receiving placebo in the core study before crossing over to lanreotide in the OLE study, AE adverse event, SD stable disease, PFS progression-free survival

Of 139 patients who were eligible for the OLE, 50 did not participate; in most cases (*n* = 37), this was because study centres did not continue participation (24 of 48 centres did not continue, mainly because lanreotide had received approval [for the symptomatic treatment of functioning NETs] in their countries and therefore, investigators did not want to participate in an open-label study). A total of 89 patients (LAN–LAN: *n* = 42; PBO–LAN: *n* = 47) entered the OLE (Fig. 1); all were included in the Safety population. The ITT population comprised 204 patients (LAN [core]: *n* = 101; PBO [core]: *n* = 103) and the PP population comprised 197 patients (LAN [core]: *n* = 96; PBO [core]: *n* = 101). Overall, 26 patients completed the OLE (were alive and had not experienced a PD event when the OLE was terminated) (Fig. 1). The maximum study duration was 8.6 years.

Baseline characteristics of the Safety population were generally similar for the LAN–LAN and PBO–LAN groups, except for WHO performance status scores (lower in the LAN–LAN group) and NET origins (in the LAN–LAN group, fewer patients had pancreatic NETs and more had hindgut NETs) (Table 1).

**Table 1:** Demographic and disease characteristics for patients participating in the OLE

	LAN–LAN group (*n* = 42)	PBO–LAN group (*n* = 47)	Total (*n* = 89)
Men	19 (45.2)	25 (53.2)	44 (49.4)
Age, mean (SD) in years^a^	64.8 (10.8)	61.3 (10.2)	62.9 (10.6)
Time since diagnosis, mean (SD) in months	36.1 (58.1)	41.8 (46.5)	39.1 (52.1)
WHO performance status score^a^			
0—normal activity	36 (85.7)	34 (72.3)	70 (78.7)
1—restricted activity	6 (14.3)	12 (25.5)	18 (20.2)
2—in bed ≤50% of the time	0	1 (2.1)	1 (1.1)
Prior NET treatment	6 (14.3)	9 (19.1)	15 (16.9)
NET origin			
Pancreas	12 (28.6)	22 (46.8)	34 (38.2)
Midgut	17 (40.5)	17 (36.2)	34 (38.2)
Hindgut	5 (11.9)	2 (4.3)	7 (7.9)
Other/unknown	8 (19.0)	6 (12.8)	14 (15.7)
Tumour progression at:			
Core study baseline	0	4 (8.5)	4 (4.5)
OLE baseline	1 (2.3)^b^	32 (68.1)	33 (37.1)
Tumour grade^c^			
G1 (Ki-67 0–2%)	30 (71.4)	32 (68.1)	62 (69.7)
G2 (Ki-67 3–10%)	12 (28.6)	15 (31.9)	27 (30.3)
Hepatic tumour load			
0%	9 (21.4)	12 (25.5)	21 (23.6)
>0–10%	19 (45.2)	19 (40.4)	38 (42.7)
>10–25%	2 (4.8)	7 (14.9)	9 (10.1)
>25–50%	10 (23.8)	5 (10.6)	15 (16.9)
>50%	2 (4.8)	4 (8.5)	6 (6.7)

Data are *n* (%), unless stated otherwise, from the Safety population and for assessments at either the CLARINET core-study baseline or ^a^CLARINET OLE baseline. Treatment groups are for patients receiving lanreotide autogel/depot 120 mg in both the CLARINET core study and the OLE (LAN–LAN group) and patients receiving placebo in the CLARINET core study and crossing over to lanreotide in the OLE (PBO–LAN group)

^b^Enroled by the investigator before communication of the results of the central assessment (PD) in the core study; patient withdrawn from the OLE on receipt of the assessment result

^c^Tumour grades based on WHO 2010 classification [15] (G1, mitotic count <2 mitoses/10 HPF and/or Ki-67 ≤ 2%; G2, mitotic count 2–20 mitoses/10 HPF and Ki-67 > 2–20%)—note that none of the patients had tumours with Ki-67 > 10%. *OLE* open-label extension, *LAN–LAN group* patients receiving lanreotide autogel/depot in core study as well as the OLE study, *PBO–LAN group* patients receiving placebo in the core study before crossing over to lanreotide in the OLE study, *WHO* World Health Organization, *NET* neuroendocrine tumour, *PD* progressive disease

Medians (ranges) for exposure to lanreotide in the Safety population were 59.0 (26.0–102.3) months in the LAN–LAN group (core study and OLE) and 23.6 (1.0–82.1) months in the PBO–LAN group (OLE only).

### Safety

The AE profile and the most common treatment-related AEs are summarised in Tables 2 and 3, respectively. In the LAN–LAN group, the overall incidences of AEs and treatment-related AEs were lower in the OLE than in the core study, and there were no clinically meaningful differences in the incidences and type of serious (SAEs) or severe AEs between the two phases (Table 2). Based on pooled data from the core study and OLE, the most common AEs were diarrhoea and abdominal pain. The incidences of these AEs were lower for the OLE compared with the core study. Treatment-related injection-site reactions occurred during the core study and OLE; the most common type of injection-site reaction was pain (Supplementary Table 1).

**Table 2:** Incidences of AEs in patients participating in the OLE according to treatment sequence

	LAN–LAN group (*n* = 42)			PBO–LAN group (*n* = 47)	
	Core study	OLE	Core study and OLE (pooled)	Core study	OLE
Any patients with an AE	39 (92.9)	34 (81.0)	40 (95.2)	44 (93.6)	42 (89.4)
Treatment-related	23 (54.8)	17 (40.5)	27 (64.3)	12 (25.5)	22 (46.8)
Severe	10 (23.8)	12 (28.6)	18 (42.9)	11 (23.4)	13 (27.7)
Moderate	19 (45.2)	16 (38.1)	17 (40.5)	25 (53.2)	22 (46.8)
Mild	9 (21.4)	6 (14.3)	4 (9.5)	8 (17.0)	7 (14.9)
Missing	1 (2.4)	0	1 (2.4)	0	0
Any patients with serious AEs	9 (21.4)	11 (26.2)	17 (40.5)	12 (25.5)	14 (29.8)
Treatment-related	1 (2.4)	2 (4.8)^a^	3 (7.1)	1 (2.1)	2 (4.3)^a^
Withdrawals due to AEs	N/A^b^	2 (4.8)^c^	2 (4.8)	N/A^b^	3 (6.4)^c^
Treatment-related	N/A^b^	0	0	N/A^b^	1 (2.1)
Most common individual AEs^d^					
Gastrointestinal disorders					
Diarrhoea	16 (38.1)	8 (19.0)	17 (40.5)	15 (31.9)	15 (31.9)
Abdominal pain	12 (28.6)	7 (16.7)	16 (38.1)	7 (14.9)	10 (21.3)
Nausea	7 (16.7)	7 (16.7)	9 (21.4)	5 (10.6)	6 (12.8)
Constipation	7 (16.7)	5 (11.9)	11 (26.2)	5 (10.6)	4 (8.5)
Vomiting	6 (14.3)	7 (16.7)	12 (28.6)	4 (8.5)	5 (10.6)
Flatulence	5 (11.9)	2 (4.8)	6 (14.3)	5 (10.6)	1 (2.1)
Abdominal distension	3 (7.1)	3 (7.1)	6 (14.3)	5 (10.6)	4 (8.5)
Dyspepsia	2 (4.8)	5 (11.9)	6 (14.3)	3 (6.4)	2 (4.3)
Upper abdominal pain	2 (4.8)	3 (7.1)	5 (11.9)	4 (8.5)	10 (21.3)
Steatorrhoea	2 (4.8)	2 (4.8)	3 (7.1)	0	5 (10.6)
Musculoskeletal and connective tissue disorders					
Arthralgia	4 (9.5)	4 (9.5)	6 (14.3)	4 (8.5)	6 (12.8)
Back pain	3 (7.1)	3 (7.1)	5 (11.9)	7 (14.9)	7 (14.9)
Musculoskeletal pain	3 (7.1)	2 (4.8)	5 (11.9)	1 (2.1)	2 (4.3)
Infections and infestations					
Nasopharyngitis	5 (11.9)	1 (2.4)	6 (14.3)	9 (19.1)	4 (8.5)
Urinary tract infection	3 (7.1)	2 (4.8)	4 (9.5)	2 (4.3)	5 (10.6)
Bronchitis	2 (4.8)	3 (7.1)	5 (11.9)	1 (2.1)	7 (14.9)
Upper respiratory tract infection	2 (4.8)	4 (9.5)	5 (11.9)	3 (6.4)	1 (2.1)
Nervous system disorders					
Dizziness	7 (16.7)	4 (9.5)	9 (21.4)	1 (2.1)	2 (4.3)
Headache	7 (16.7)	2 (4.8)	9 (21.4)	6 (12.8)	4 (8.5)
General disorders and administration site conditions					
Fatigue	4 (9.5)	5 (11.9)	8 (19.0)	6 (12.8)	4 (8.5)
Asthenia	7 (16.7)	0	7 (16.7)	5 (10.6)	4 (8.5)
Metabolism and nutrition disorders					
Decreased appetite	5 (11.9)	4 (9.5)	6 (14.3)	3 (6.4)	5 (10.6)
Hyperglycaemia	3 (7.1)	3 (7.1)	6 (14.3)	0	1 (2.1)
Skin and subcutaneous tissue disorders					
Rash	4 (9.5)	5 (11.9)	6 (14.3)	2 (4.3)	2 (4.3)
Psychiatric disorders					
Insomnia	2 (4.8)	4 (9.5)	6 (14.3)	1 (2.1)	0
Vascular disorders					
Hypertension	6 (14.3)	5 (11.9)	10 (23.8)	3 (6.4)	5 (10.6)
Respiratory, thoracic and mediastinal disorders					
Oropharyngeal pain	3 (7.1)	3 (7.1)	5 (11.9)	0	2 (4.3)
Hepatobiliary disorders					
Cholelithiasis	6 (14.3)	9 (21.4)	14 (33.3)	4 (8.5)	7 (14.9)

Data are number (%) of patients with an AE, while receiving lanreotide autogel/depot 120 mg or placebo and are from the Safety population. AEs were defined according to the MedDRA version 18.1

*AE* adverse event, *OLE* open-label extension, *LAN–LAN* *group* patients receiving lanreotide autogel/depot in core study as well as the OLE study, *PBO–LAN* group patients receiving placebo in the core study before crossing over to lanreotide in the OLE study, *N/A* not applicable, *MedDRA* Medical Dictionary for Regulatory Activities

^a^LAN–LAN group: two patients experienced cholelithiasis; PBO–LAN group: one patient experienced tumour necrosis and one experienced pancreatitis

^b^N/A not applicable (patients who were withdrawn from the core study because of AEs were not eligible for inclusion in the OLE)

^c^LAN–LAN group: 1 patient with ileus and 1 with evolving stroke (fatal); PBO–LAN group: 1 patient with sudden death, 1 with a fall (fatal), and 1 with tumour necrosis (also reported as a serious AE) and tumour haemorrhage (tumour necrosis and haemorrhage were reported to be treatment related)

^d^Based on MedDRA version 18.1 preferred terms; AEs occurring in ≥10% of patients in any group

**Table 3:** Incidences of the most common (occurring in ≥5% of patients) treatment-related AEs in patients participating in the OLE according to treatment sequence

	LAN–LAN group (*n* = 42)			PBO–LAN group (*n* = 47)	
	Core study	OLE study	Both studies (pooled)	Core study	OLE study
Diarrhoea	12 (28.6)	4 (9.5)	13 (31.0)	4 (8.5)	12 (25.5)
Abdominal pain	7 (16.7)	0	7 (16.7)	1 (2.1)	1 (2.1)
Cholelithiasis	4 (9.5)	7 (16.7)	10 (23.8)	2 (4.3)	6 (12.8)
Hyperglycaemia	3 (7.1)	1 (2.4)	4 (9.5)	0	1 (2.1)
Flatulence	3 (7.1)	1 (2.4)	3 (7.1)	2 (4.3)	0
Injection-site pain	3 (7.1)	1 (2.4)	4 (9.5)	1 (2.1)	3 (6.4)
Steatorrhoea	2 (4.8)	2 (4.8)	3 (7.1)	0	4 (8.5)
Injection-site nodule	1 (2.4)	0	1 (2.4)	0	3 (6.4)

Data are number (%) of patients with an AE and are from the Safety population. AEs were defined according to the MedDRA version 18.1

*AE* adverse event, *OLE* open-label extension, *LAN–LAN group* patients receiving lanreotide autogel/depot in core study as well as the OLE study, *PBO–LAN group* patients receiving placebo in the core study before crossing over to lanreotide in the OLE study, *MedDRA* Medical Dictionary for Regulatory Activities

Comparing the PBO–LAN and LAN–LAN groups, there were no clinically meaningful differences in the overall incidences of AEs, treatment-related AEs, severe AEs or SAEs during the OLE (Table 2). In the PBO–LAN group, the incidence of diarrhoea irrespective of causality was similar between the core study and the OLE (Table 2). The time course of diarrhoea AEs is summarised in Supplementary Table 2a. Most cases of diarrhoea in lanreotide autogel/depot-treated patients occurred during the first 12–24 weeks of treatment. Treatment-related cholelithiasis was reported in ten patients (23.8%) in the LAN–LAN group and eight patients (17.0%) in the PBO–LAN group (data from core study and OLE); in the PBO–LAN group, six of the eight cases occurred during the OLE. Most cases of cholelithiasis occurred after week 36 of LAN treatment (Supplementary Table 2b). In addition, most cholelithiasis AEs were mild to moderate in severity and none resulted in treatment discontinuation or withdrawal from the study. The duration of abdominal pain events by primary tumour location is summarised in Supplementary Table 3. The majority of events were in patients with midgut and pancreatic tumours (both *n* = 32), followed by other/unknown (*n* = 8) and hindgut (*n* = 2), with a median duration of 25.5 days, 17.5 days, 10.5 days and 271.0 days, respectively.

There was one serious, treatment-related case of cholelithiasis (in the OLE in the LAN–LAN group); the patient was hospitalised, but recovered without any action being taken. There were two additional SAEs during the OLE that were considered treatment-related by investigators: one case of (severe) tumour necrosis and one case of (moderate) pancreatitis, both in the PBO–LAN group. The sponsor considered that the case of tumour necrosis (accompanied by tumour haemorrhage) was most likely due to the underlying disease, as this can be a pathological feature of NETs with liver metastases [7]. The case of pancreatitis, which was due to biliary obstruction, lasted 6 days and the patient recovered. No action was taken with the study treatment, i.e., it was not interrupted or discontinued as a result of the event. Only one patient was withdrawn from the study because of treatment-related AEs (this was the patients with tumour necrosis and tumour haemorrhage).

For the gallbladder echography data of the 47 patients with data and no lithiasis at OLE baseline, 4 patients in each group (9.5% and 8.5% of patients in the LAN–LAN and PBO–LAN group, respectively) had developed lithiasis by the time of the last visit. Of the 48 patients with data and no gallbladder sludge at OLE baseline, sludge developed by the time of the last visit in 2 (4.8%) patients and 1 (2.1%) patient in the LAN–LAN and PBO–LAN group, respectively.

There were small mean changes in haematology and biochemistry laboratory parameters, including HbA1c, between baseline and the last visit of the OLE, but these were generally considered to be not clinically relevant. Mean values for all assessed ECG parameters showed mild fluctuations above and below baseline values, but remained generally consistent throughout the study.

### Efficacy

In the LAN–LAN group, one patient died (as a result of stroke-in-evolution) and 18 had PD; one of the 18 patients with PD died as a result, but only the PD event was included in the PFS analyses (Fig. 1). In the PBO–LAN group, 3 patients died and 27 had PD; of the 27 PD events, 7 were first PD events (including 1 with PD at the final study visit) and 20 were subsequent PD events (Fig. 1).

**Fig. 2:**
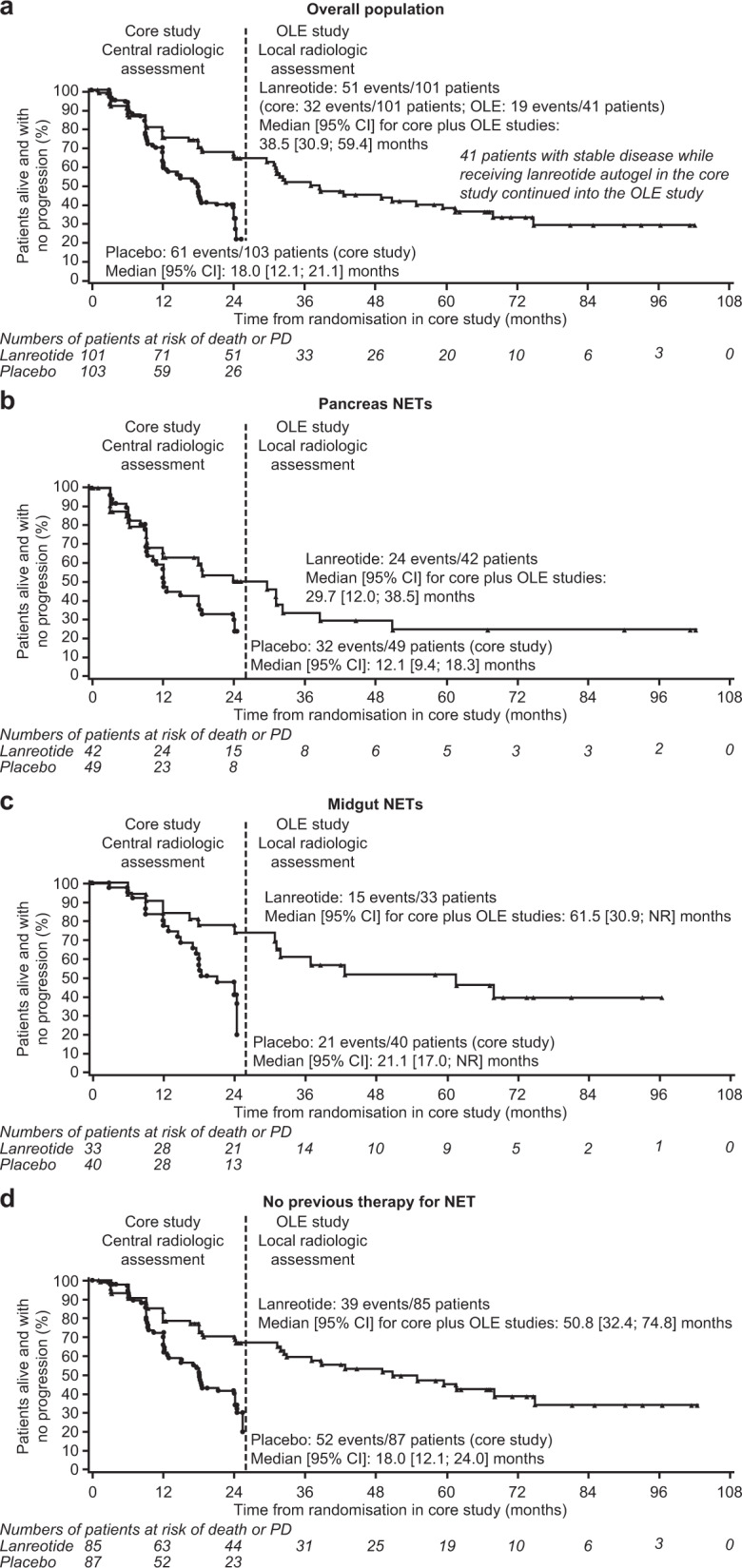
PFS for lanreotide autogel/depot from the CLARINET core study and the OLE and PFS for placebo from the core study: overall (**a**) and for subgroups according to primary tumour origin (**b**, **c**) and prior therapy (**d**). Events were PD (according to RECIST version 1.0) or death. Data are from the intention-to-treat population with months approximated based on 4 weeks per month. Core-study data are from all patients randomly allocated to double-blind treatment (lanreotide autogel/depot or placebo). The OLE data are only for patients originally randomly allocated to lanreotide in the core study who then continued into the OLE. The PFS data previously reported for placebo were based on 60 events at the time of database lock in the core study [1]; however, one patient with PD had been erroneously reported as having centrally assessed SD. This additional event been included in the analysis of the OLE data. For the pancreas and midgut data, primary tumour type is the basis for the analyses and results are based on the combination of the various primary tumour locations. OLE open-label extension, PD progressive disease, NET neuroendocrine tumour, NR not reached, PFS progression-free survival, RECIST Response Evaluation Criteria In Solid Tumours, SD stable disease

In the LAN–LAN group (i.e., data from the OLE final analysis appended to data from the core study), the median [95% CI] PFS for lanreotide was 38.5 [30.9; 59.4] months (Fig. 2). Sixteen of the 41 eligible patients enroling in the OLE (i.e., excluding the patient enroled with PD) and continuing to receive lanreotide completed the extension without PD (Fig. 1).

Results for the supportive and sensitivity analyses were generally consistent with the main efficacy endpoint. Median [95% CI] PFS in the PP population was 37.1 [30.9; 59.4] months. In the a priori sensitivity analysis to evaluate the potential impact of patient withdrawal due to investigator assessment of PD despite central assessment of SD (*n* = 15 patients), the median [95% CI] PFS was 32.4 [24.0; 50.8] months. In the *post-hoc* sensitivity analysis of potential selection bias due to 25 patients completing the core study (13 in the lanreotide group and 12 in the placebo group) with SD but not continuing into the OLE (which assumed these patients had PD at the first OLE assessment), the median [95% CI] PFS was 30.8 [30.0; 37.1] months in the LAN–LAN group and 18 [12.1; 24.0] months in the PBO–LAN group. In addition, PFS curves in the clinically relevant subgroups were generally consistent with the ITT analysis, although some subgroups were very small (Table 4 and Fig. 2).

**Table 4:** PFS for lanreotide autogel/depot 120 mg from the CLARINET core study and the OLE and PFS for placebo from the core study across subgroups defined according to baseline characteristics in the core study

Subgroup	Number of events/patients		Median PFS [95% CI] (months)	
	LAN (core and OLE) *N* = 101	PBO (core) *N* = 103	LAN (core and OLE)	PBO (core)
Tumour origin				
Midgut	15/33	21/40	61.5 [30.9, NR]	21.1 [17.0, NR]
Pancreas	24/42	32/49	29.7 [12.0, 38.5]	12.1 [9.4, 18.3]
Hindgut	5/11	2/3	55.0 [2.9, NR]	24.4 [12.0, 24.4]
Other/unknown	7/15	6/11	59.4 [32.8, 74.8]	15.0 [6.3, NR]
Tumour grade^a^				
G1 (Ki-67 0–2%)	32/69	41/72	50.8 [31.3, 74.8]	18.2 [12.1; 24.0]
G2 (Ki-67 3–10%)	19/32	19/29	31.2 [16.6, 32.8]	12.1 [9.0; 18.0]
Missing	0/0	1/2	–	–
Hepatic tumour load				
≤25%	28/62	42/75	50.8 [31.3, 74.8]	18.6 [17.0; 24.4]
>25%	23/39	19/28	24.1 [9.3, 49.0]	9.4 [6.3; 12.0]
Progressive disease at baseline of core study				
Yes	3/4	3/5	3.1 [3.0, 3.2]	6.2 [3.0, NR]
No	48/97	58/98	38.7 [31.2, 61.5]	18.0 [12.1, 21.1]
Previous therapy for non-functioning NET				
Yes^b^	12/16	9/16	29.7 [6.0, 31.3]	12.0 [3.3, NR]
No	39/85	52/87	50.8 [32.4, 74.8]	18.0 [12.1, 24.0]
Geographical region				
USA	6/16	9/14	61.5 [12.0, NR]	9.4 [9.0, NR]
Outside of the USA	45/85	52/89	37.1 [29.7, 55.0]	18.0 [12.1, 24.0]

*PFS* progression-free survival, *OLE* open-label extension, *LAN* lanreotide autogel/depot 120 mg, *PBO* placebo, *NET* neuroendocrine tumour, *NR* not reached, *SD* stable disease

^a^Tumour grades based on WHO 2010 classification [15] (G1, mitotic count <2 mitoses/10 HPF and/or Ki-67 ≤ 2%; G2, mitotic count 2–20 mitoses/10 HPF and Ki-67 > 2–20%)—note that none of the patients had tumours with Ki-67 > 10%;

^b^Number of patients who received previous chemotherapy: LAN *n* = 14, PBO *n* = 15; Yttrium (90Y) compounds: LAN *n* = 4, PBO *n* = 0; proton pump inhibitors: LAN *n* = 2, PBO *n* = 2; octreotide: LAN *n* = 2, PBO *n* = 1; interferons, LAN *n* = 1, PBO, *n* = 1; monoclonal antibodies, LAN *n* = 1, PBO *n* = 0; opioids: LAN *n* = 0, PBO *n* = 2 (patients could have more than one previous therapy). Data are from the intention-to-treat population with months approximated based on 4 weeks per month and were for subgroups defined a priori except for tumour grade and hepatic tumour load. Core-study data are from all patients randomly allocated to double-blind treatment (lanreotide autogel/depot or placebo). The OLE data are only for patients originally randomly allocated to lanreotide in the core study who then continued into the OLE. The PFS data previously reported for placebo were based on 60 events overall [1]; this was because 1 patient was erroneously reported as having centrally assessed SD at the time of database lock in the core study. This has been revised in the analysis of the OLE data.

**Fig. 3:**
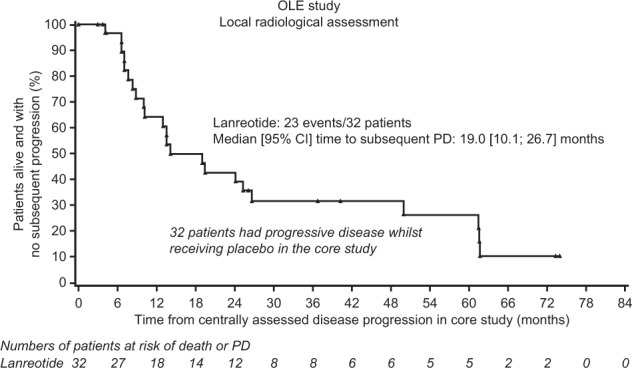
Time to death or subsequent PD in patients with PD, while receiving placebo in the core study who switched to lanreotide autogel/depot in the OLE. Data are from a subset of the intention-to-treat population with months approximated based on 4 weeks per month. OLE open-label extension, PD progressive disease

In the PBO (PD)–LAN group, the median [95% CI] time from first PD event (while receiving placebo during the core study) to death or subsequent PD (while receiving open-label lanreotide) was 19 [10.1; 26.7] months (Fig. 3). Of the 32 patients in the OLE who had previously experienced PD while receiving placebo during the core study, 3 (9.4%) completed the study without a subsequent event, 3 (9.4%) died and 20 (62.5%) experienced a subsequent PD (Fig. 1).

Of the 15 patients in the PBO–LAN group who entered the OLE with SD from the core study, seven (46.7%) experienced PD events during the OLE (there were no deaths); median [95% CI] PFS was 47.0 [6.0; not reached (NR)] months. Amongst these 15 patients, one (who experienced a PD event during the OLE) did not complete the core study before enroling into the OLE (Fig. 1). Median PFS for the 14 patients with SD at the end of the core study who continued in the OLE was not reached.

## Discussion

The final analyses from the OLE of the CLARINET study provide new data on the safety and efficacy of lanreotide autogel/depot 120 mg every 4 weeks in patients with non-functioning metastatic enteropancreatic NETs. Only one additional patient was included after the interim analysis was conducted because the date of database lock was the same for the CLARINET core study and OLE interim analysis; however, the median (range) duration of lanreotide autogel/depot 120 mg treatment was much longer in the final OLE analysis: 59 (26.0–102.3) months (core and OLE combined), compared to 40 (26.0–74.3) in the interim analysis [4] and 24 (1.0–25.3) months in the core study [1]. The most commonly reported AEs were diarrhoea, abdominal pain and cholelithiasis, and AE data during the OLE were in line with those reported during the core study, confirming that lanreotide autogel/depot given at 120 mg every 4 weeks is generally well tolerated during chronic treatment. The favourable safety and tolerability profile of lanreotide in CLARINET OLE is consistent with the results from other clinical trials of lanreotide in patients with NETs [8–10] and from studies conducted in everyday clinical practice worldwide over many years [11, 12].

In terms of efficacy, the final results of the OLE study provide a median [95% CI] PFS for lanreotide of 38.5 [30.9; 59.4] months. This contrasts with the median [95% CI] PFS for placebo of 18.0 [12.1–24.0] months reported in the core study [1]. Thus, collectively, the safety and efficacy data support the early and long-term use of lanreotide autogel/depot 120 mg for enteropancreatic NETs. The OLE also provided data for placebo-treated patients who had progressed during the core study; median [95% CI] time to death or subsequent PD in these patients was 19 [10.1; 26.7] months. Although these data are uncontrolled and based on a smaller number of patients, they are nevertheless clinically important in view of the relative lack of data on the effect of SSAs in patients with PD. In the CLARINET core study, only 4% of patients had documented progression (according to RECIST v1.0) before inclusion in the study [1]; this likely reflects a lack of data at the time of enrolment into CLARINET on the effect of SSAs in patients with PD and reluctance on the part of investigators to potentially administer placebo to these patients. Arguably, the PBO–LAN population in CLARINET OLE may not fully reflect the original PBO population in CLARINET as they may be considered to have been on an active surveillance period; however, our results in the patient population included in the OLE (38% of whom had pancreatic NETs and 38% of whom had NETs of the small intestine or appendix) suggest that a delay in further tumour progression can be expected in lanreotide-treated patients with PD. Other SSA studies in progressive NETs have provided similar results to those obtained in the CLARINET OLE. In a phase II study (also uncontrolled) in 30 patients with progressive NETs (gastrointestinal 47%, pancreatic 27% and lung 13%) treated with lanreotide autogel/depot 120 mg every 4 weeks for up to 92 weeks, PFS was 12.9 months [10]. In a post-hoc analysis of data from the RADIANT-2 study, median PFS in treatment-naive patients with progressive NETs was 13.6 months after initiation of octreotide long-acting release [13].

The extended exposure to lanreotide autogel/depot 120 mg and longer follow-up in the final CLARINET OLE dataset also facilitated analyses in clinically relevant subgroups. In the LAN–LAN group, PFS results across subgroups based on tumour origin, grade and hepatic tumour load, PD and previous therapy status at core study baseline, and region (US/non-US) were generally consistent with the main analysis. Of note, median [95% CI] PFS for lanreotide was 61.5 [30.9; NR] months in patients with midgut primary tumours, 55 [2.9, NR] months in those with hindgut primary tumours and 29.7 [12.0; 38.5] months in patients with pancreatic primary tumours. Comparing hepatic tumour load, median [95% CI] PFS was 50.8 [31.3, 74.8] and 24.1 [9.3, 49.0] for ≤25% vs >25%, respectively. In addition, median [95% CI] PFS was 50.8 [32.4; 74.8] months in the subgroup not receiving previous therapy for non-functioning NETs, compared with 29.7 [6.0; 31.3] months for patients receiving previous therapy, although the number of patients in the latter group was small (*n* = 16). The final analysis of CLARINET OLE describes the longest PFS for these subgroups, to date. Previous studies have also identified differences in PFS among patient subgroups. Palazzo et al. [12] performed multivariate analyses on subgroups of lanreotide-treated patients with malignant digestive NETs, which revealed significant associations between PFS and patients with a proliferation index (Ki-67) of ≤5% (*p* = 0.009), pre-treatment tumour stability (*p* = 0.008), or hepatic tumour load of ≤25% (*p* = 0.004). Similarly to the tumour grade subgroup results observed in the present study (G1: 50.8 [31.3, 74.8] versus G2: 31.2 [16.6, 32.8] months), Faggiano et al. [14] showed a longer PFS survival in SSA-treated patients with G1 compared with G2 gastro-entero-pancreatic or thoracic NETs, although this difference was not significant (89 versus 43 months, respectively; *p* = 0.15). However, when differences were assessed between patients with a Ki-67 index of <5% compared with >5%, those with a Ki-67 index <5% had a significantly longer PFS (89 compared with 35 months, respectively; *p* = 0.005) [14].

One caveat associated with interpretation of the PFS data relates to the small number of patients receiving lanreotide during the core study who did not continue through to the OLE despite being eligible (*n* = 13); this was because some study centres did not participate in the OLE. This was addressed in a *post-hoc* sensitivity analysis, which assumed that these patients had an event at the first scheduled radiological assessment in the OLE. The results were not dissimilar to those of the main analysis, providing a median [95% CI] PFS of 30.8 [30.0; 37.1] months.

Limitations of the CLARINET OLE study include the lack of control group and the fact that scans were obtained only every 6 months and were assessed by local review. Nevertheless, the estimate of median PFS for lanreotide autogel/depot 120 mg was based on the ITT population from the core study and mainly on events confirmed centrally. Another limitation of CLARINET OLE is that some eligible patients did not take part, due to non-participation of some of the study centres that participated in the core study. However, as discussed above, this was addressed in a *post-hoc* sensitivity analysis, the results of which were not substantially different from those of the main analysis. This sensitivity analysis took the most conservative approach to the ‘missing’ patients, assuming that all had disease progression at the first follow-up visit in the OLE.

In conclusion, results from the CLARINET OLE study provide new evidence for the long-term safety of lanreotide autogel/depot in indolent and progressive metastatic intestinal or pancreatic NETs, demonstrating a safety and tolerability profile that is consistent with the results of previous trials, including the CLARINET core study. The results of this study also indicate that long-term treatment with lanreotide autogel/depot 120 mg every 4 weeks has sustained anti-tumour effects in patients with non-functioning metastatic grade 1 or 2 (Ki-67 < 10%) enteropancreatic NETs, irrespective of tumour origin. This is based on the PFS results obtained in patients treated with lanreotide for a median of ~5 years and up to 8.6 years. Based on data from placebo-treated patients with PD in the core study, the results also demonstrate that the anti-tumour effects of lanreotide extend to patients with progressive NETs. Together with the acceptable safety and tolerability profile, these data indicate that lanreotide autogel/depot should be initiated early at a dose of 120 mg every 4 weeks in patients with enteropancreatic NETs and continued long-term in these patients.

## Supplementary information

Supplementary Information
