# Supplementary material for: Lanreotide autogel/depot in advanced enteropancreatic neuroendocrine tumours: final results of the CLARINET open-label extension study
Source: Endocrine. 2020 Oct 14;71(2):502–13. doi: 10.1007/s12020-020-02475-2 (PMC7881960; doi:10.1007/s12020-020-02475-2)
Supplement: Supplementary file 1 — Supplementary Information [file 12020_2020_2475_MOESM1_ESM.docx]

**Supplementary Appendix**

**Protocol amendments occurring during the study**

The protocol was finalised on 4 September 2008. Protocol amendments 1–3, which occurred prior to the planned interim analysis, were described previously [4]. Subsequent amendments are described below.

Protocol amendment 4 (22 October 2013)

- Study conduct: to increase the maximum expected study duration to approximately 8 years
- Analyses: to confirm that a second interim analysis was to be performed to be able to provide a safety updated report during the review procedure
- Administrative: change due to the transfer of pharmacovigilance activities to the sponsor

Protocol amendments 5 and 6 (19 June 2014 and 9 April 2015, respectively)

- Administrative: changes to personnel and contact details

**CLARINET core study and OLE investigators**

| **Investigator** | **Affiliation** |
| --- | --- |
| M. Raderer | European Neuroendocrine Tumor Society (ENETS) Center of Excellence, Medical University of Vienna, Vienna, Austria |
| I. Borbath* | Department of Gastroenterology and Digestive Oncology, Cliniques Universitaires Saint-Luc and Université Catholique de Louvain, Brussels, Belgium |
| D. Ysebaert* | Department of Hepatobiliary, Transplantation and Endocrine Surgery, University Hospital Antwerp, Edegem, Belgium |
| E. Sedláčková* | Department of Oncology of the First Faculty of Medicine and General Teaching Hospital, Prague, Czech Republic |
| P. Vítek* | Proton Therapy Center Czech, Prague, Czech Republic |
| H. Grønbæk | Department of Hepatology and Gastroenterology, Aarhus University Hospital, Aarhus, Denmark |
| P. Holeckova* | Department of Oncology and Radiotherapy, Hospital Na Bulovce and 1st Medical Faculty of Charles University, Prague, Czech Republic |
| A. Adenis* | Département d'Oncologie Médicale, Institut du Cancer de Montpellier, Montpellier, France |
| L. Buscail | Department of Gastroenterology and Pancreatology, University of Toulouse, oulouse, France |
| G. Cadiot* | Service d'Hépato gastroentérologie, Hopital R.Debré, Reims, FRANCE |
| S. Dominguez* | Département de cancérologie digestive et urologique, CAC Oscar Lambret, Lille, France |
| M. Ducreux | Gustave Roussy, Department of Medical Oncology, Villejuif, France; Université Paris-Saclay, France |
| C. Lombard-Bohas* | Unité d'oncologie Médicale, Hôpital Edouard Herriot, Lyon, France |
| E. Mitry | Département d'oncologie médicale, Institut Curie, Ensemble hospitalier, Hôpital René Huguenin, Saint-Cloud, France |
| P. Ruszniewski* | Division of Gastroenterology and Pancreatology, Beaujon Hospital, Clichy, and Université de Paris, Paris, France |
| J.F. Seitz | CHU la Timone, Service de gastro entérologie, Aix-Marseille-University, Marseille, France |
| F. El Hajbi* | Département de cancérologie digestive et urologique, CAC Oscar Lambret, Lille, France |
| N. Begum | Chirurgische Klinik, Universitätsklinikum, Lübeck, Germany |
| I. Harsch | Department of Internal Medicine II, Thuringia Clinic "Georgius Agricola", Saalfeld/Saale, Germany. |
| M. Pavel | Department of Medicine, Division of Endocrinology and Diabetology, Universitätsklinikum Erlangen, Friedrich Alexander University Erlangen-Nürnberg, Erlangen, Germany |
| C. Schöfl | Medizinische Klinik I mit Poliklinik, Universitätsklinikum, Erlangen, Germany |
| M. Weber | Klinik u. Poliklinik für Innere Med. Endokrin. u. Stoffwechsel, Klinikum der Johannes-Gutenberg-Universität, Mainz, Germany |
| B. Wiedenmann | Hepatologie u. Gastroenterologie Charité, Campus Virchow Klinikum, Berlin, Germany |
| M. Mallath | Department of Digestive Disease and Clinical Nutrition, TATA Memorial Centre, Maharashtra, India |
| P. Patil* | Department of Digestive Disease and Clinical Nutrition, TATA Memorial Centre, Maharashtra, India |
| K. Sambasivaiah | Department of Medical Oncology, TATA Memorial Centre, Maharashtra, India |
| R. Saxena* | Medical Oncology Department, Global Hospitals, Hyderabad, India |
| E. Bajetta* | Institute of Oncology, Policlinico di Monza, Monza, Italy |
| A. Buonadonna* | Department of Medical Oncology, Unit of Medical Oncology & Cancer Prevention, Centro di Riferimento Oncologico di Aviano, Aviano, Italy |
| R. Buzzoni* | Istituto Nazionale per lo Studio e la Cura dei Tumori, Oncologia Medica B,  Milano, Italy |
| R. Cannizzaro* | Centro di Riferimento Oncologico, Oncologia Medica C, Aviano (PD), Italy |
| A. Colao* | Dipartimento di Endocrinologia e Oncologia, Università di Napoli, Federico II, Molecolare e Clinica, Napoli, Italy |
| C. De Angelis* | Gastroenterology and Hepatology Unit, Città della Salute e della Scienza, Turin, Italy |
| P. Tomassetti | Unità Operativa Chirurgia Generale, Azienda Ospedaliera Policlinico S. Orsola-Malpigh, Bologna; Italy |
| J. Ćwikła* | Diagnostic and Therapeutic Center – Gammed, Warsaw, Poland |
| B. Kos‑Kudła | Department of Endocrinology and Neuroendocrine Tumors, ENETS Center of Excellence, Department of Pathophysiology and Endocrinology, Medical University of Silesia, Katowice, Poland |
| T. Salek* | Department of Biomedical Sciences, Medical Faculty, University of Ostrava, Ostrava, The Czech Republic |
| J. Capdevila* | Servicio de Oncología, Hospital Universitari Vall d'Hebron, Barcelona, Spain |
| G. Soler | Servicio de Oncología Médica, Institut Català d´Oncologia, Hospital Duran i Reynals, Barcelona, Spain |
| J.M. Tabernero | Servicio de Oncología, Hospital Universitari Vall d'Hebron, Barcelona, Spain |
| A. Teulé Vega* | Servicio de Oncología Médica, Institut Català d´Oncologia, Hospital Duran i Reynals, Barcelona, Spain |
| H. Ahlman | Goteborg University, Sahlgrenska Hospital, Surgery Department, Goteborg, Sweden |
| M. Kjellman | Department of Breast and Endocrine Surgery, Karolinska University Hospital, Stockholm, Sweden |
| G. Aithal* | Queens Medical Centre, University Hospitals of Nottingham, Nottingham, UK |
| A. Anthoney | St James Institute of Oncology, University of Leeds, Leeds, UK |
| M. Caplin* | Department of Gastroenterology and Tumour Neuroendocrinology, Royal Free Hospital, London, UK |
| A. Grossman | Centre for Endocrinology, Barts and the London School of Medicine, Queen Mary University of London, London, UK |
| J. Newell-Price | Department of Oncology and Metabolism, University of Sheffield. Sheffield. UK |
| J. Ramage | Department of Gastroenterology, Basingstoke and North Hampshire Hospital, Hampshire, UK |
| N. Reed* | Beatson West of Scotland Cancer Centre, Glasgow, UK |
| A. Rees* | Neuroscience & Mental Health Research Institute, University Hospital of Wales, Cardiff, UK |
| W. Steward | University Department of Oncology, Leicester Royal Infirmary, Leicester, UK |
| L. Wall* | Edinburgh Cancer Centre, Western General Hospital. Edinburgh, UK |
| M. Choti* | Department of Surgery, Banner MD Anderson Cancer Center, Gilbert, AZ, USA |
| A.T. Phan | Department of Hematology‐Oncology, University of Texas Health Science Center at Tyler, Tyler, TX, USA |
| E.M. Wolin* | Center for Carcinoid and Neuroendocrine Tumors, Mount Sinai Hospital, New York, NY, USA |
| T. Pawlik* | The Ohio State University, Wexner Medical Center, Columbus, OH, USA |
| A. Hendifar* | Cedars-Medical Center, Samuel Oschin Comprehensive Cancer Institute, Los Angeles, CA, USA |

*CLARINET OLE Investigator.

**Supplementary Table 1. Treatment-related injection-site reactions in patients participating in the OLE according to treatment sequence**

|  | **LAN–LAN group (*n* = 42)** | |  | **PBO–LAN group (*n* = 47)** |
| --- | --- | --- | --- | --- |
|  | **OLE study** | **Core study + OLE (pooled)** |  | **OLE study** |
| Injection site |  |  |  |  |
| Pain | 1 (2.4) | 4 (9.5) |  | 3 (6.4) |
| Pruritus | 2 (4.8) | 2 (4.8) |  | 1 (2.1) |
| Rash | 0 | 1 (2.4) |  | 0 |
| Mass | 1 (2.4) | 1 (2.4) |  | 1 (2.1) |
| Granuloma | 0 | 1 (2.4) |  | 0 |
| Haematoma | 1 (2.4) | 1 (2.4) |  | 0 |
| Nodule | 0 | 1 (2.4) |  | 3 (6.4) |
| Reaction | 0 | 1 (2.4) |  | 0 |
| Induration | 0 | 0 |  | 2 (4.3) |

Data are number (%) of patients with an AE and are from the Safety population. Adverse events were defined according to the Medical Dictionary for Regulatory Activities version 18.1. *LAN* lanreotide autogel/depot 120 mg, *OLE* open-label extension, *PBO* placebo

**Supplementary Table 2. Time course of (A) diarrhoea events and (B) cholelithiasis events**

**(A)**

| **Time at onset (weeks)** | **LAN–LAN (*n* = 42)** | | **PBO–LAN (*n* = 47)** | |
| --- | --- | --- | --- | --- |
|  | Core | OLE | Core | OLE |
| 0–12 | 12 (28.6) | 1 (2.4) | 6 (12.8) | 11 (23.4) |
| >12–24 | 5 (11.9) | 0 | 2 (4.3) | 2 (4.3) |
| >24–36 | 1 (2.4) | 1 (2.4) | 2 (4.3) | 1 (2.1) |
| >36–48 | 1 (2.4) | 3 (7.1) | 4 (8.5) | 0 |
| >48–72 | 5 (11.9) | 3 (7.1) | 2 (4.3) | 3 (6.4) |
| >72–96 | 3 (7.1) | 2 (4.8) | 4 (8.5) | 2 (4.3) |
| >96–120 | – | 2 (4.8) | – | 1 (2.1) |
| >120–144 | – | 1 (2.4) | – | 1 (2.1) |
| >144–168 | – | 1 (2.4) | – | 0 |
| >168 | – | 4 (9.5) | – | 0 |

(B)

| **Time at onset (weeks)** | **LAN–LAN (n=42)** | | **PBO–LAN (n=47)** | |
| --- | --- | --- | --- | --- |
|  | Core | OLE | Core | OLE |
| 0–12 | 1 (2.4) | 0 | 1 (2.1) | 0 |
| >12–24 | 0 | 0 | 0 | 0 |
| >24–36 | 0 | 1 (2.4) | 0 | 1 (2.1) |
| >36–48 | 0 | 0 | 0 | 2 (4.3) |
| >48–72 | 4 (9.5) | 3 (7.1) | 3 (6.4) | 2 (4.3) |
| >72–96 | 0 | 0 | 0 | 1 (2.1) |
| >96–120 | 1 (2.4) | 0 | 0 | 3 (6.4) |
| >120–144 | – | 2 (4.8) | – | 0 |
| >144–168 | – | 0 | – | 0 |
| >168 | – | 4 (9.5) | – | 0 |

*LAN* lanreotide autogel/depot 120 mg, *OLE* open-label extension, *PBO* placebo

**Supplementary Table 3. Duration (in days) of abdominal pain events by location (pooled data from core and OLE study)**

| **Primary tumour location** | **LAN–LAN (*n* = 42)** | **PBO–LAN (*n* = 47)** | **Total (*n* = 89)** |
| --- | --- | --- | --- |
| Midgut  N  Mean (SD)  Median (Q1 – Q3)  Min – Max | 19  196.1 (434.3)  31.0 (14.0 – 131.0)  1.0 – 1864.0 | 13  81.5 (253.1)  6.0 (5.0 – 19.0)  1.0 – 923.0 | 32  149.5 (370.9)  25.5 (5.0 – 70.5)  1.0 – 1864.0 |
| Hindgut  N  Mean (SD)  Median (Q1 – Q3)  Min – Max | 1  409.0 ( – )  409.0 (409.0 – 409.0)  409.0 – 409.0 | 1  133.0 ( – )  133.0 (133.0 – 133.0)  133.0 – 133.0 | 2  271.0 (195.2)  271.0 (133.0 – 409.0)  133.0 – 409.0 |
| Pancreas  N  Mean (SD)  Median (Q1 – Q3)  Min – Max | 7  138.9 (254.2)  15.0 (5.0 – 156.0)  2.0 – 701.0 | 25  133.5 (330.9)  20.0 (5.0 – 65.0)  1.0 – 1594.0 | 32  134.7 (311.9)  17.5 (5.0 – 78.5)  1.0 – 1594.0 |
| Other/unknown  N  Mean (SD)  Median (Q1 – Q3)  Min – Max | 5  70.4 (114.2)  12.0 (9.0 – 57.0)  3.00 – 271.0 | 3  27.0 (37.4)  9.0 (2.00 – 70.0)  2.0 – 70.0 | 8  54.1 (91.4)  10.5 (6.0 – 63.5)  2.0 – 271.0 |

Abdominal pain events includes the following preferred terms: ‘Abdominal pain’, ‘abdominal pain upper’ and ‘abdominal pain lower’, defined according to the Medical Dictionary for Regulatory Activities (version 18.1). *LAN* lanreotide autogel/depot 120 mg, *OLE* open-label extension, *PBO* placebo, *SD* standard deviation, *Q* quartile.
